# Society Meeting

**Published:** 1915-11

**Authors:** 


### SOCIETY MEETING

**The Medical Society of the County of Ontario,** following the state law, has appointed a County Milk Commission. The medical profession is represented by Dr. A. L. Beahan, chairman, and Dr. Wm. A. King.

